# Förster Resonance Energy Transfer (FRET) Correlates of Altered Subunit Stoichiometry in Cys-Loop Receptors, Exemplified by Nicotinic α4β2

**DOI:** 10.3390/ijms130810022

**Published:** 2012-08-10

**Authors:** Rahul Srinivasan, Christopher I. Richards, Crystal Dilworth, Fraser J. Moss, Dennis A. Dougherty, Henry A. Lester

**Affiliations:** 1 Division of Biology, California Institute of Technology, Pasadena, CA 91125, USA; E-Mails: rahul@caltech.edu (R.S.); cir@caltech.edu (C.I.R.); crystal@caltech.edu (C.D.); fraserjmoss@gmail.com (F.J.M.); 2 Division of Chemistry and Chemical Engineering, California Institute of Technology, Pasadena, CA 91125, USA; E-Mail: dadoc@caltech.edu

**Keywords:** nicotine, cytisine, NFRET, nicotine addiction, Parkinson’s disease, ion channels

## Abstract

We provide a theory for employing Förster resonance energy transfer (FRET) measurements to determine altered heteropentameric ion channel stoichiometries in intracellular compartments of living cells. We simulate FRET within nicotinic receptors (nAChRs) whose α4 and β2 subunits contain acceptor and donor fluorescent protein moieties, respectively, within the cytoplasmic loops. We predict FRET and normalized FRET (NFRET) for the two predominant stoichiometries, (α4)_3_(β2)_2_ *vs*. (α4)_2_(β2)_3_. Studying the ratio between FRET or NFRET for the two stoichiometries, minimizes distortions due to various photophysical uncertainties. Within a range of assumptions concerning the distance between fluorophores, deviations from plane pentameric geometry, and other asymmetries, the predicted FRET and NFRET for (α4)_3_(β2)_2_ exceeds that of (α4)_2_(β2)_3_. The simulations account for published data on transfected Neuro2a cells in which α4β2 stoichiometries were manipulated by varying fluorescent subunit cDNA ratios: NFRET decreased monotonically from (α4)_3_(β2)_2_ stoichiometry to mostly (α4)_2_(β2)_3_. The simulations also account for previous macroscopic and single-channel observations that pharmacological chaperoning by nicotine and cytisine increase the (α4)_2_(β2)_3_ and (α4)_3_(β2)_2_ populations, respectively. We also analyze sources of variability. NFRET-based monitoring of changes in subunit stoichiometry can contribute usefully to studies on Cys-loop receptors.

## 1. Introduction

A superfamily of ligand-gated ion channels comprises homo- and heteropentameric combinations of subunits. The superfamily includes neuronal nicotinic acetylcholine receptors (nAChRs) comprised of α (α2 to α10) and β (β2 to β4) subunits. Possible changes in the stoichiometry of these subunits within the α4β2 pentamer have generated interest, because the (α4)_2_(β2)_3_ and (α4)_3_(β2)_2_ stoichiometries differ in their sensitivity to agonists [1,2] and to antagonists [2], their rectification properties [3], their Ca^2+^ permeability [4], their sensitivity to upregulation by chronic nicotine and other drugs [5,6], and possibly their subcellular localization. Some of these characteristics may provide signatures for determining the stoichiometry of nAChRs when they appear on the plasma membrane. However, several nAChRs and other Cys-loop receptors localize to the endoplasmic reticulum (ER) [6–8], where stoichiometry measurements with biochemical methods are either tedious [5] or impossible. Therefore it is necessary to have additional robust measurements of changes in intracellular receptor stoichiometry.

Chronic nicotine increases the assembly of (α4)_2_(β2)_3_ receptor pentamers within the ER [6,9], enhances the ER exit of nAChRs, and therefore upregulates plasma membrane receptors [6]. Such selective intracellular pharmacological chaperoning of acetylcholine receptor number and stoichiometry explains several aspects of nAChR upregulation by nicotine and other nicotinic ligands [6,9–15]. In addition to the obvious connection with nicotine addiction, pharmacological chaperoning may provide the mechanistic basis for the inverse correlation between a person’s history of smoking and his/her susceptibility to Parkinson’s disease [12]. These points increase our interest in intracellular measurements of alterations in α4β2 nAChR stoichiometry.

We and others have utilized fluorescent protein (FP)-tagged α4 and β2 nAChR subunits and fluorescence microscopy in transfected cells to study altered α4β2 stoichiometry [6,16–19]. This paper provides the theoretical basis of an improved FRET method for monitoring these changes. In our previous experiments, we incorporated donors in 50% of the α subunits, and acceptors in the 50% of α subunits, leaving the β subunits unlabelled, or vice-versa. In our more recent experiments, all β subunits contained donors, and all α subunits contain acceptors, or vice-versa [6]. In this case, (1) in a pentamer with three acceptors and two donors, FRET is stronger than in the two acceptor-three donor case; and (2) FRET is also stronger between adjacent than between non-adjacent subunits. The key prediction is the ratio of the FRET for the stoichiometries, (α4)_3_(β2)_2_ *vs*. (α4)_2_(β2)_3_. We show that the new method does not require additional strong assumptions about symmetry, or about distances between fluorophores. The method may be considered a special case within the tradition of analyzing pentameric proteins by FRET [20–23]. The theory is of interest because the measurements have yielded estimates about changes in stoichiometry that are consistent with known structural information and with chaperoning by nicotine, as well as a novel effect of chaperoning by cytisine.

## 2. Results and Discussion

### 2.1. Simulations

**Figure 1:**
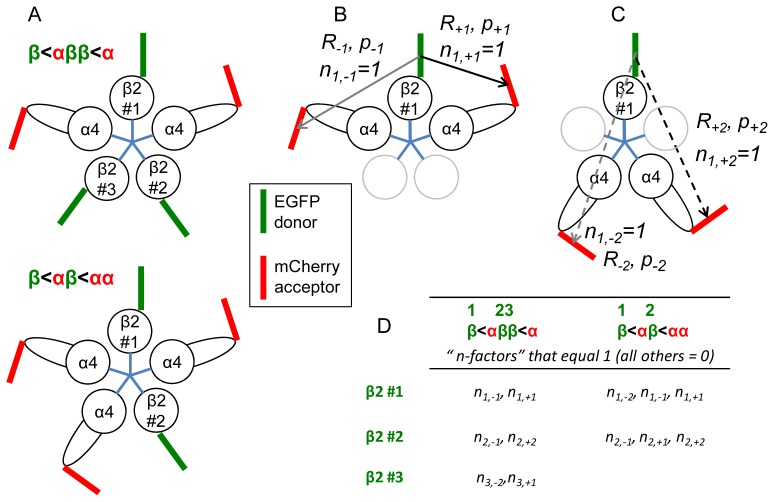
Diagrams depicting FRET analysis of nAChR stoichiometry. (**A**) Two examples of stoichiometry, thought to correspond to the major α4β2 nAChRs in brain [12]. Both stoichiometries contain two β2 < α4 interfaces. The < character implies a correctly polarized high-affinity ligand binding interface, contrasting with the arrows in (**B**) and (**C**) which denote energy transfer. The nAChR is viewed from the extracellular surface. In the top panel the stoichiometry is (α4)_2_(β2)_3_, and in the bottom panel (α4)_3_(β2)_2_. The donor molecules are labeled 1 through 3, as in the equations in the text; (**B**) Nomenclature for energy transfer to acceptors that are adjacent to the donor. The first subscript denotes the identity of the β2-EGFP donor subunit. The second subscript is ±1 for adjacent and non-adjacent energy transfer, respectively; the positive sign denotes clockwise transfer. β2-EGFP donor #1 is shown with two immediately flanking α4-mCherry acceptors. Because these two pathways exist, the “*n*-factors” *n*_1, −1_ = *n*_1, +1_ = 1. The arrows show the fluorophore separations *R*_±1_, *R*_±2_ and the corresponding energy transfer probabilities, *p*_±1_, *p*_±2_ Clockwise energy transfer is shown as a black arrow; anticlockwise, as gray. The remaining two subunits are shown in gray; they may be either donors or acceptors; (**C**) Nomenclature for energy transfer to acceptors that are non-adjacent to the donor. β2-EGFP donor #1 is shown with two α4-mCherry acceptors that are non-adjacent to the donor (but incidentally adjacent to each other). Because these two pathways exist, the “*n*-factors” *n*_1, −2_ = *n*_1, +2_ = 1. Other details as in (**B**); (**D**) Table showing the “*n*-factors” for the nAChR stoichiometries under consideration. See text.

We calculate the relative FRET efficiency *(E)* for nAChRs composed of the (α4-mCherry)_3_(β2-EGFP)_2_ *vs*. (α4-mCherry)_2_(β2-EGFP)_3_ stoichiometry. We define the average FRET values as *Ē*_3,2_ and *Ē*_2,3_ respectively, so that the desired ratio is *Ē*_3,2_ / *Ē*_2,3_. We then extend these calculations to NFRET and calculate the analogous ratios, *NFRET*_3,2_/*NFRET*_2,3_. The assumptions provide a special case of previous theories about pentameric proteins, but are less restrictive than previous analyses of pentameric ion channels [18,21], as described below (see Figure 1).

(a) All β2-EGFP subunits have identical structure, and all α4-mCherry subunits have identical structure. For simplicity, we generally omit the description of the fluorophore when discussing stoichiometry; thus in most cases (α4)_2_(β2)_3_ implies (α4-mCherry)_2_(β2-EGFP)_3_.

(b) FRET interactions are fully determined by relative positions of subunits within the pentamer. The EGFP donor fluorophores need not be located at the same radial distance as the mCherry acceptors. The donor fluorophores also need not have the same dipole moment angle with the radius, nor lie in the same plane, as the acceptors. Indeed, the intracellular domain of the wild-type α4 subunit has roughly twice as many amino acids as that of the wild-type β2 subunit, rendering it likely that the α4-linked fluorophores have a different disposition from β2-linked fluorophores.

(c) Donor subunits can be either adjacent or non-adjacent to acceptor subunits. Figure 1A depicts a receptor with (α4)_2_(β2)_3_ and (α4)_3_(β2)_2_ stoichiometry in the upper and lower panels respectively, and also shows the nomenclature for distinguishing among the three or two donor fluorophores, so that calculations can proceed for each donor. Figure 1A also shows the usual assumption, that two interfaces exist in which the β subunit is immediately counterclockwise to the α subunit. These appropriately polarized interfaces, which we term β2 < α4, are important for function because they form high-affinity agonist/antagonist binding sites; however the presence of a ligand site does not affect the FRET properties (the analogous terminology applied to GABA_A_ or GluCl receptors would be α<β, because for such receptors, the principal interface for binding lies on the β subunit). The fluorophores are shown as green (EGFP donor) and red (mCherry acceptor) bars, to emphasize the importance of the fluorophores’ dipoles. The locations and orientations of the dipoles are arbitrary. The wild-type α4 M3–M4 intracellular loop has a sequence roughly twice as long the wild-type β2 subunit; this loop is depicted schematically, and the EGFP donor fluorophore projects from the loop. Figure 1A thus emphasizes that the donor and acceptor fluorophores can also differ with respect to their radial distance. The donors can also reside in a different plane from the acceptors; this point is not shown. The distances between donor and acceptor fluorophores, angle between the donor emission and acceptor absorption dipoles, and spectral overlap integrals, lead to four possible microscopic FRET probabilities *p_j_* for each donor. These are calculated as described below. Figure 1B depicts the energy transfer pathways when a donor is flanked by adjacent acceptors; and Figure 1C shows the pathways when two acceptors are both non-adjacent to a donor. Depending on the order and stoichiometry of the subunits, only specific subsets of these pathways exist for each donor. Figure 1D tabulates the “*n*-factors” for the nAChR stoichiometries under consideration. The subunit order is clockwise, seen from the extracellular surface. The two or three β2-EGFP subunits are numbered, as in A. The first column corresponds to A, top panel; the second, to A, bottom panel. Each cell in the table reports *n_i,j_* values that equal 1 (all others = 0), corresponding to possible energy transfer pathways. The *n_i,j_* values control the appearance of terms in Equations 2 through 6.

For Equations (1) through 8 below, we consider only those donor and acceptor molecules within assembled receptors. The rate constant τ*_DA,i_*^−1^ for decay from the excited state increases linearly with the contribution from each potential donor-acceptor interaction:

(1) $$\tau_{DA,i}^{-1}=\tau_{D}^{-1}(1+f_{P}\sum_{j}n_{i,j}p_{j})=\tau_{D}^{-1}(1+f_{P}\sum_{j}n_{i,j}\frac{R_{j,0}^{6}}{R_{j}^{6}})$$

where τ*_D_*^−1^ is the unperturbed rate constant. The equations select among the available energy transfer pathways as follows: each *p_j_* is multiplied by a parameter that we term an “*n*-factor” *n_i,j_*. The index *i* describes one of the two (*i* = 1, 2) or three (*i* = 1, 2, 3) individual donor fluorophores in the pentamer, and *j* = −2, −1, +1, +2 describe acceptors located at adjacent (±1), non-adjacent (*±*2) positions, where negative numbers indicate anticlockwise transfer as described in Figure 1B,C. The “*n*-factor” *n_i,j_* is unity if donor *i* has an acceptor at relative position *j*, and zero if not. For example, if there is an α4-mCherry acceptor immediately clockwise to β2-EGFP donor #2, then *n*_2, +1_ = 1; and the associated *p*_+1_ therefore exists in the term describing β2-EGFP donor #2 in Equations 2, 3, 9, and 10 below. Figure 1D is a table of all “*n*-factors” that equal unity.

Measured spectral FRET values always return products of the Förster efficiency E multiplied by the fraction *f_P_* of fluorophores participating in the energy transfer within assembled pentamers. The parameter is less than unity because donor and/or acceptor fluorophores fold incompletely, mature incompletely, become proteolyzed, undergo partial bleaching, or otherwise fail to participate in FRET [24]. The parameter *f_P_* is included in Equations 2 and 3 below. The possibility of unpaired, free donors residing outside of pentamers is considered later.

In Equation 1, the FRET probabilities are calculated, as usual, by terms of the form $p_{j}=\frac{R_{j,0}^{6}}{R_{j}^{6}}$. *R_j_*_,0_ is the Förster distance for each *j-*valued donor-acceptor pair, and *R_j_* is the distance between each *j-*valued donor-acceptor pair (length of the arrow in Figure 1). The Förster distance is determined by the spectral overlap between EGFP and mCherry (this is constant among all *j-*valued pairs) and by the dipole angle between donor and acceptor; the latter parameter determines the orientation factor *κ*^2^, which potentially varies among the *j* values. For the specific assumption that *κ*^2^ = 2/3, the Förster radius *R*_0_ for the EGFP-Cherry pair is 51.4 Å [25]. However, we do not explicitly assume that the fluorophores move so quickly that they sample many orientations; indeed, this assumption is unacceptable for fluorescent proteins embedded within a loop. The parameter Δ, described below, embodies the possibility that *κ*^2^ differs among donor-acceptor pairs within a pentamer.

We now compute the desired ratio, *E*_3, 2_/*E*_2, 3_. The (α4)_2_(β2)_3_ nAChR stoichiometry of Figure 1A, top panel, has 3 donor molecules:

(2) $$\begin{array}{c} \tau_{DA,1}^{-1}=\tau_{D}^{-1}\left[1+f_{P}(p_{+1}+p_{-1})\right],\;\;\;\tau_{DA,2}^{-1}=\tau_{D}^{-1}[(1+f_{P}(p_{+1}+p_{-2})],\\ \tau_{DA,3}^{-1}=\tau_{D}^{-1}[1+f_{P}(p_{-1}+p_{+2})] \end{array}$$

In the (α4)_3_(β2)_2_ configuration of Figure 1A, lower panel, the two donors have

(3) $$\tau_{DA,1}^{-1}=\tau_{D}^{-1}[1+f_{P}(p_{+1}+p_{-1}+p_{+2})],\;\;\;\tau_{DA,2}^{-1}=\tau_{D}^{-1}[1+f_{P}(p_{+1}+p_{-1}+p_{-2})]$$

We now apply the equation,

(4) $$E_{i}=1-\frac{\tau_{DA,i}}{\tau_{D}}$$

We average over the two donors or the three donors, as appropriate (all conventional optical methods perform such averaging, because the resolution is much poorer than the subunit spacing). Thus,

(5) $$\bar{E}(3,2)=\bar{1-\frac{\tau_{DA,i}}{\tau_{D}}}=1-\frac{1}{2}\left[\frac{1}{1+f_{P}(p_{+1}+p_{-1}+p_{+2})}+\frac{1}{1+f_{P}(p_{+1}+p_{-1}+p_{-2})}\right]$$

and

(6) $$\bar{E}(2,3)=1-\frac{1}{3}\left[\frac{1}{1+f_{P}(p_{+1}+p_{-1})}+\frac{1}{1+f_{P}(p_{+1}+p_{-2})}+\frac{1}{1+f_{P}(p_{-1}+p_{+2})}\right]$$

These relations allow us to calculate *Ē*_3,2_ / *Ē*_2,3_.

**Figure 2:**
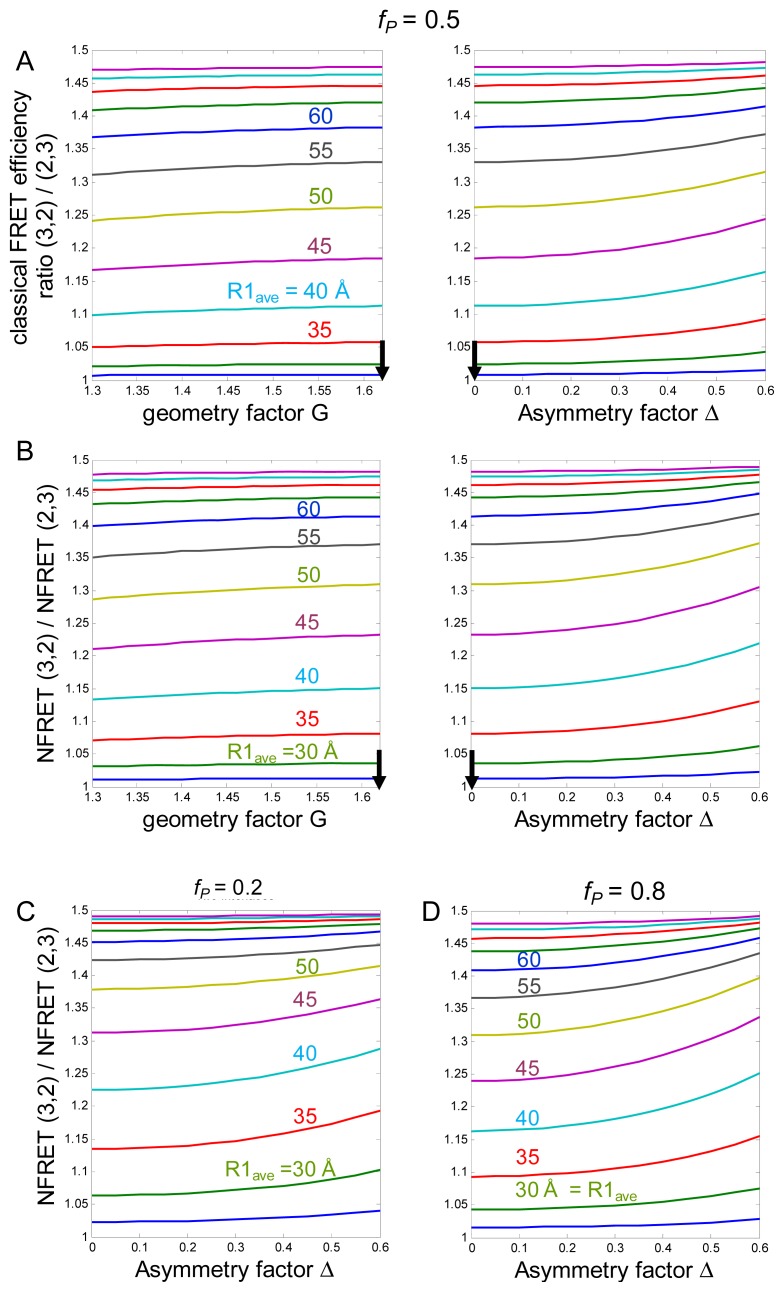
Simulations. (**A**) The *y*-axis is the simulated *Ē*_3,2_ / *Ē*_2,3_*,* the ratio of classical FRET efficiency for the two stoichiometries. The vertical arrows give parameters associated with the regular pentagonal receptor. The theoretical curves are computed for various values of the average distance between fluorophores on adjacent subunits *R_1,ave_*. *X*-axes are (**left**) the geometry factor G, and (**right**) the asymmetry factor Δ. The assumed *f_P_* = 0.5; (**B**) Same parameters as in (**A**); the plotted values are *NFRET*_3,2_/*NFRET*_2,3_, the ratio of NFRET efficiency for the two stoichiometries; (**C**) (**D**) Values of *NFRET*_3,2_/*NFRET*_2,3_ *vs*. asymmetry factor Δ for assumed *f_P_* = 0.2 (**C**) or 0.8 (**D**).

(d) An unknown value is the distance between fluorophores on adjacent subunits, because there are no structural data for the cytoplasmic M3–M4 loops. We define an average distance *R*_1,_*_ave_* between fluorophores on adjacent subunits. We then calculate the quantities of interest for a range of *R*_1,_*_ave_* values. The sensitivity of FRET measurements is limited to distances within a factor of two of *R_0_*. The lower limit of our simulations, 25 Å, represents both ~ *R_0_*/2 and the closest possible distance between adjacent fluorophores of the GFP family. The upper limit corresponds to ~2*R_0_* = 100 Å. As discussed below (Figure 2), the *Ē*_3,2_ / *Ē*_2,3_ and *NFRET*_3,2_/*NFRET*_2,3_ ratios both approach the theoretical limit of 1.5 at ~80 Å, obviating exploration of greater *R*_1,_*_ave_* values.

In order to perform specific calculations in the absence of structural data about the M3–M4 loops, we further define a regular pentagon-shaped receptor, as well as variations around this standard, as follows.

(e) If the five fluorophores are located at the vertices of a pentagon, we may write,

(7) $$R_{+1}=R_{-1}=R_{1,ave},\;\;\;R_{+2}=R_{-2}=R_{2,ave}={GR}_{1,ave}$$

We define a “geometry factor”, *G*, which equals 1.62 (the ratio of the length of a diagonal to a side of a pentagon) for the pentagonal receptor. Any departure from a regular plane pentagonal structure, or any difference between the plane of the α4-mCherry *vs*. β2-EGFP fluorophores [26], would decrease G. Figure 2B shows how the calculated *Ē*_3,2_/*Ē*_2,3_ values vary for *G* between 1.3 and 1.62.

(f) We define an “asymmetry factor”, Δ, that can arise from unequal *R_j_* values, and/or of *κ*^2^ values, between the donor and two possible flanking acceptors that are both adjacent to the donor (as in Figure 1B). For simplicity, we allow the same parameter to represent the asymmetry for the two possible acceptors that are non-adjacent to the donor (as in Figure 1C).

$$p_{+1}(1+\Delta )p_{1,ave},\;p_{-1}=(1-\Delta )p_{1,ave},\;p_{+2}=(1+\Delta )p_{2,ave},\;p_{-2}=(1-\Delta )p_{2,ave}$$

The special case of Δ = 0 defines a regular pentameric, pentagonal receptor: (i) the five fluorophores are located at 72° intervals with respect to the nAChR axis of pseudo symmetry, presumably the channel pore; and (ii) their dipole moments make a common angle with the radius. In the simulations, we allow the range Δ = 0 to 0.6 (the latter value indicates that *p*_+1_ differs from *p*_+1_/*p*_−1_ ~3 *p*_−1_ by 4-fold). Figure 1B shows an extreme case in which the difference between *R*_−1_ and *R*_+1_ produces *p*_+1_/*p*_−1_ slightly greater than the 4-fold range. Monte Carlo calculations on a regular pentagon (Figure 10C of Reference [21]) show that *κ*^2^ between two adjacent subunits lies mostly within a range of ±10%; the *κ*^2^ values between non-adjacent subunits are somewhat larger, but again lie mostly within a range of ±10%. The corresponding range is *p*_+1_/*p*_−1_ ~3.

These relations lead to Figure 2A, which plots *Ē*_3,2_ / *Ē*_2,3_ *versus G* and Δ; this curve is repeated at 5 Å intervals of *R*_1,_*_ave_*. Our experiments have used sensitized emission measurements, in which FRET is assessed by acceptor fluorescence [6,18,27]. The average total fluorescence from a given pentamer is the average of the energy transferred via resonance, times the quantum yield of the acceptor fluorophores *Q_Ai_*:

$$\bar{I_{DA,i}}=Q_{A}\bar{1-\frac{\tau_{DA,i}}{\tau_{D}}}$$

Thus, the *Q_Ai_* factor cancels out, and the equations for sensitized emission FRET are the right-hand sides of Equations 5 and 6. We also define *f_D_*^′^, the fraction of donors that actually reside in fully assembled pentamers (including fully assembled, but immature, non-glycosylated or post-translationally modified pentamers). The remaining donors would reside in FRET-incapable soluble proteins or in partially FRET-capable, partially assembled receptors. The prime notation reminds us that FRET capability is additionally represented, within assembled pentamers, by *f_P_*. Assuming that *f_D_*^′^ does not depend on stoichiometry, this factor does not contribute to the FRET ratio calculations for sensitized emission.

#### 2.1.1. Calculations of NFRET

Most of our experiments use NFRET, a variant in which the sensitized emission is normalized as follows:

(8) $$NFRET=\frac{I_{DA}}{\sqrt{I_{D}}\sqrt{I_{A}}}$$

In Equation 8,*I_A_* is the fluorescence from all acceptor molecules, both those that receive energy from donors and those that do not [24]. To the extent that *I_A_* is measured accurately by direct excitation at the excitation wavelength for the acceptor, *I_A_* does not depend explicitly on the stoichiometry and therefore cancels out when one takes the ratio *NFRET*_3,2_/*NFRET*_2,3_. In Supplementary Material, we analyze possible deviations from this assumption.

In Equation 8,*I_D_* is the donor fluorescence. This fluorescence is reduced because some EGFP molecules undergo FRET within pentamers; but EGFP molecules outside pentamers have much less or zero FRET. Therefore we write,

(9) $$I_{D}=c\left(1-f_{D}^{\prime }\bar{E}\right)$$

where *c* is a multiplicative constant which cancels out when ratios are computed. *I_D_* decreases to zero if both *Ē* =1 and *f_D_*^′^ =1. Our simulations use *f_D_*^′^ =1, because this is the most “pessimistic” assumption from the viewpoint that NFRET is a reasonable monitor of FRET. With these assumptions, NFRET is approximately proportional to FRET efficiency *E* for *E* < ~0.75, but NFRET becomes infinite as *E* approaches unity [28]. This poorly behaved range is not approached in our simulations, for three reasons. First, *f_P_* remains <1. Thus, in our previous studies, the maximal FRET efficiency was 48% [17,18,29]. Second, *R*_1,_*_ave_* remains ≥ *R_0_*/2. Third, the process of computing *NFRET*_3,2_/*NFRET*_2,3_ cancels out additional poorly behaved characteristics of *NFRET*. As a result, Figure 2 reveals that *NFRET*_3,2_/*NFRET*_2,3_ varies in much the same way as *Ē*_3,2_ / *Ē*_2,3_ over a wide range of simulated parameters, as long as *f_P_* < ~0.8 (*NFRET*_3,2_/*NFRET*_2,3_ does become poorly behaved for *f_P_* *>* ~0.9). Our techniques do not require that NFRET remains strictly proportional to FRET over the simulations, or that *NFRET*_3,2_ / *NFRET*_2,3_ = *Ē*_3,2_ / *Ē*_2,3_*,* but simply that *NFRET*_3,2_/*NFRET*_2,3_ lies in a useful range for all plausible values of nAChR structure.

Measurements of net FRET are treated in Supplementary Data. We found measurements of net FRET less useful those of NFRET.

#### 2.1.2. Summary of the Predictions

We emphasize the major predictions that 1 / 1.5 < *Ē*_3,2_ *Ē*_2,3_ < and that 1 < *NFRET*_3,2_/*NFRET*_2,3_ < 1.5 for all reasonable structures of α4β2 nAChRs. The limit of 1.0 at small values of *R*_1,_*_ave_* occurs because the energy transfer approaches equal efficiency for all donors, regardless of the number of acceptors. The limit of 1.5 at large *R*_1_, arises from the fact that the average β2-EGFP donor has 1.5 times as many adjacent α4-mCherry acceptors in the (α4)_3_(β2)_2_ stoichiometry as in the (α4)_2_(β2)_3_ stoichiometry; and at distances much greater than *R_0_*, the adjacent subunits dominate the energy transfer (*p_±_*_1,_ *p_±2_*). The quantitative predictions are rather more sensitive to variations in the asymmetry factor Δ than in the geometry factor G (Figure 2).

### 2.2. Agreement with Biased Transfection Experiments

We have utilized nAChR α4-mCherry (acceptor) and β2-EGFP (donor) subunits. These fluorophores have, in our hands, optimal characteristics for pixel-by-pixel sensitized emission in living cells. These proteins are expressed in Neuro-2a cells, a favorable system in which membrane protein expression remains linear with respect to several factors under the experimenter’s control [6,16–19]. In one set of experiments, Neuro-2a cells were transfected with 0.33, 0.5, or 0.67 mole fraction cDNAs for the β2-EGFP subunit (the balance was α4-mCherry cDNA, for a total of 1 μg plasmid DNA) [27]. These give more robust signals than in our previous studies utilizing only fluorescent α subunits, or only fluorescent β subunits. Measurements based on individual pixels (rather than on the image from an entire cell) make the best use of available data from confocal imaging [30–32]. For instance, despite the substantial pixel-to-pixel variance, the mean NFRET values have standards errors of the mean much less than 1%. Sensitized emission measurements are nondestructive and can therefore be repeated many times on a single cell. NFRET equals *I_DA_* normalized to the square root of the donor × acceptor intensity and is readily comparable between different samples [33]. The simulations (Figure 2) show that NFRET ratios provide measures that are similar to *Ē*_3,2_ / *Ē*_2,3_ ratios. We have therefore used pixel-by-pixel sensitized emission NFRET as the most appropriate parameter to monitor changes in subunit stoichiometry [6,18,19,27].

**Figure 3:**
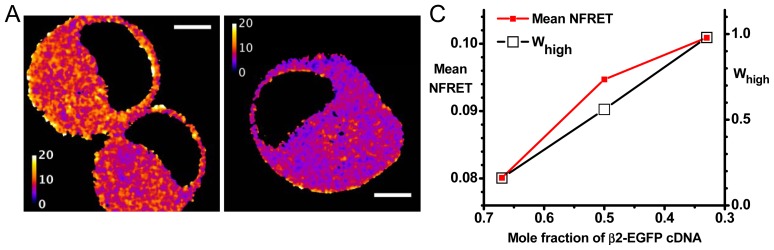

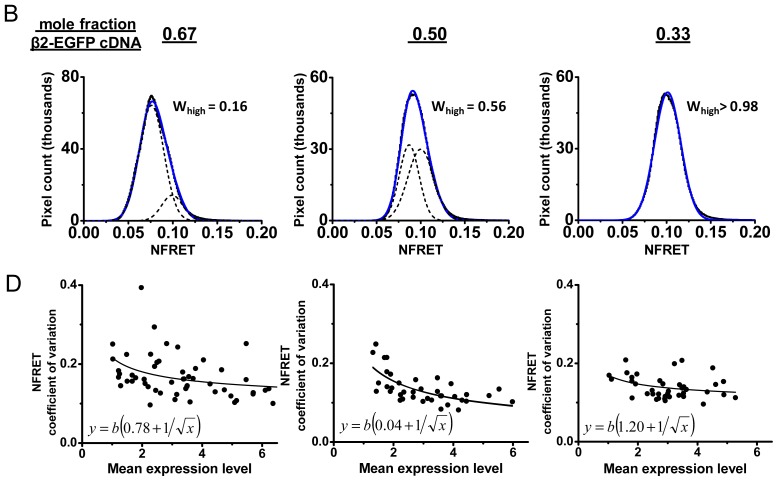
Data from NFRET measurements with biased transfection ratios. (**A**) NFRET images for representative cells transfected with 0.5 mole fraction plasmid ratios of β2-EGFP and α4-mCherry. The lookup table runs from 0 to 0.2 fractional NFRET. Scale bars, 5 μm; (**B**) NFRET data for transfection with 0.67, 0.5, and 0.33 mole fraction plasmid ratios of β2-EGFP. For each graph, the heavy line is the measured histogram; this is generally obscured by the blue curve which sums the Gaussians fitted to the high and low NFRET components (dashed lines). The computed fractional area of high NFRET pixels (*W_high_*) is shown in each case; (**C**) Average NFRET and *W_high_* *vs*. mole fraction of β2-EGFP cDNA transfected, from the data in (**B**). The left *y*-axis gives mean NFRET. The standard errors of the mean are much smaller than the size of symbols, because each sample had > 10^6^ pixels. The right *y*-axis is *W_high_*; (**D**) Plots of the CV of the NFRET pixel distribution (*y*-axis) *vs*. the expression level (*x*-axis). Each point corresponds to a cell. Expression level is quantified as NFRET normalization, the mean of $\left(\sqrt{I_{eGFP}}\sqrt{I_{mcherry}}\right)$ for each cell. The fitted curves have the form, $y=b(a+\sqrt{x})$.

Figure 3 presents further analysis of our most complete experiment, previously published as supplementary information [27]. For the experiment of Figure 3, when the β2-EGFP mole fraction was 0.67 and 0.33, the average NFRET was 0.0798 and 0.101, respectively. We tentatively assume that these ratios produce nearly pure populations of (α4)_2_(β2)_3_ and (α4)_3_(β2)_2_ nAChRs, respectively (this assumption will be explored further in the next section). These data yield / 1.27 *Ē*_3,2_ *Ē*_2,3_ =. Thus, the data confirm that the NFRET approach can differentiate between the (α4)_2_(β2)_3_ and (α4)_3_(β2)_2_ receptor stoichiometries. Figure 2A indicates that the data would be satisfied by *R_1,ave_* values between 35 and 50 Å, depending on the assumptions for G and Δ. Our analyses assume the α4, β2 subunit order in which there are two β2<α4 interfaces (Figure 1A). The data would also be consistent with a single-interface model (see Supplementary Material).

#### 2.2.1. Further Analysis Based on Distinct Populations

We wish to detect changes in the fractions of receptors with each stoichiometry, utilizing the full power of analyzing each pixel. The most appropriate way to analyze NFRET distributions uses Gaussian components [19,22,30]. We note that two Gaussian components are required to fit the pixel-by-pixel distribution of NFRET in most cells. The average NFRET values differ among cells transfected in the same dish, and also between subcellular regions of individual cells. These characteristics can be observed in Figure 3. At the subcellular level, these variations exceed those observed for intracellular images of soluble proteins undergoing FRET [28]. Previous studies suggest that these variations arise from the fact that the nAChRs are localized primarily on organelle membranes [6]. To characterize the distribution of FRET values among pixels, but without committing to a particular source of the variation, we pool the entire distribution of pixels in the entire collection of 30–55 cells under each condition [6,19,31]. The predicted $\bar{NFRET_{3,2}}/\bar{NFRET_{2,3}}<1.5$, so that one does not expect to observe two well-resolved peaks; and we found no such case. We therefore fitted pixelby- pixel NFRET histograms to two Gaussian components, with peak (=average) values denoted $\bar{NFRET_{high}}$ and $\bar{NFRET_{low}}$.

Trends similar to Figure 3A–C were also observed in two less complete experiments. The average ratio, $\bar{NFRET_{high}}/\bar{NFRET_{low}}$ in all experiments was 1.23 and the full range of measured ratios was 1.18 to 1.25. Thus the NFRET distributions display peaks corresponding to the extremes of $\bar{NFRET}$ that we measured by biasing the cDNA ratios. If the NFRET values for the pure stoichiometries, *NFRET*_3,2_ and *NFRET*_2,3_, are greater and less than *NFRET_high_* and *NFRET_low_*, respectively, we would expect the NFRET distributions to include peaks or shoulders at these extreme values, outside the range defined by the averages for the biased cDNA ratios. However the distributions reveal no such extreme components. These arguments provide confidence that the *NFRET_high_* and *NFRET_low_* components correspond to the (α4)_3_(β2)_2_ and (α4)_2_(β2)_3_ stoichiometries.

We calculate the integrals, *Int_low_* and *Int_high_*, of the *NFRET_low_* and *NFRET_high_* component in each case. We then calculate the weighted contribution of the *Int_high_* component:

(10) $$W_{high}=Int_{high}/(Int_{low}+Int_{high})$$

Cells transfected with a mole fraction of 0.5 β2-EGFP cDNA subunits and 0.5 α4-mCherry showed a nearly equal proportion of high and low NFRET distributions (*W_high_* = 0.56). For cells expressing 0.67 and 0.33 mole fraction of β2-EGFP cDNA, *W_high_* was 0.16 and >0.98 respectively (Figure 2). While there is no straightforward way to provide error estimates for *W_high_*, note that we require a coefficient of determination value of 0.995 to accept the number of components (one or two) in the fitted distributions [6,27]. The idea that a pure (α4)_3_(β2)_2_ population forms more readily than a pure (α4)_2_(β2)_3_ population agrees with recent data from stably transfected cell lines [34]. Thus we conclude that *W_high_* increases monotonically, but perhaps not linearly, with the fraction of (α4)_3_(β2)_2_ stoichiometry. Therefore changes in *W_high_* denote changes in the fraction of α4β2 nAChRs with the two stoichiometries.

#### 2.2.2. Variations in Stoichiometry among Pixels and among Cells

**Figure 4:**
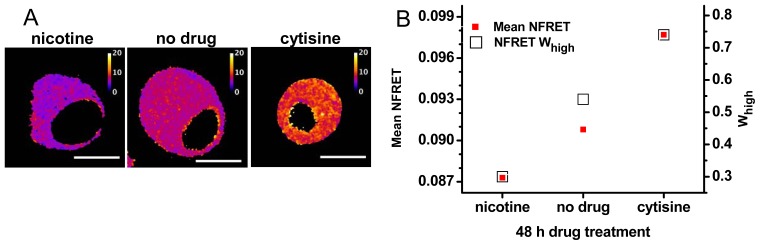
Data from NFRET measurements with pharmacological chaperones. Further analysis of a published experiment [27]. (**A**) NFRET images for representative cells transfected with 0.5 mole fraction plasmid ratios of β2-EGFP and α4-mCherry. Incubation for 48 h in 0.1 μM nicotine (left), no added drug (center), and 0.1 μM cytisine (right). The lookup table runs from 0 to 0.2 fractional NFRET. Scale bars, 10 μm; (**B**) Average NFRET and *W_high_* for each incubation, from data like those of Figure 1B. The left *y*-axis gives mean NFRET. The standard errors of the mean are much smaller than the size of symbols, because each sample had >10^6^ pixels. The right *y*-axis is *W_high_*.

The formal Gaussian fits in our experimental data (Figures 3 and 4) [6,27] could arise either if (a) each component contains a mixture of individual pixels, each containing a pure (α4)_3_(β2)_2_ or (α4)_2_(β2)_3_ population, or (b) individual pixels contain a mixture of (α4)_3_(β2)_2_ or (α4)_2_(β2)_3_. The subcellular mechanisms that partially segregate these populations are not yet clear. At present our ability to distinguish among these is limited by variability in FRET measurements arising in part from optical distortions [32].

NFRET serves well to compare data among cells with varying expression levels. Nonetheless, a cell with a higher expression level is expected to provide a less noisy overall estimate of FRET. Suppose that the number of nAChR molecules undergoing FRET in a given pixel is a uniformly distributed random variable whose mean varies among cells. (A cell is also a collection of compartments that may have their own means; NFRET in intracellular compartments has been reported separately [27]). Measurements from a uniformly distributed collection of molecules are expected to have standard deviation proportional to the square root of the average number of molecules. In this paper, we extend the previous analyses by comparing the distributions across cells by plotting the coefficient of variation (CV) of the NFRET signal *vs*. the expression level. We approximated the expression level within a cell by taking either its mean net FRET or its mean normalization value (denominator in Equation 8); results were similar for the two metrics, and the second is presented in Figure 3D. In the data for 0.5 mole fraction β2-EGFP cDNA, we found that the CV varies as the −0.48 power of expression level, which is near the expected inverse square-root relation. Figure 4D displays further analyses for mole fractions of 0.33, 0.5, and 0.67 β2-EGFP cDNA. We fitted the distributions to the sum of a constant term and an inverse square-root term, $y=b(a+\sqrt{x})$. The small *a* value in the middle plot indicates that the CV shows a nearly ideal inverse square-root dependence on the expression level. The larger *a* value in the first and third panels show that CV has an additional constant component. The inverse square-root term represented nearly the entire distribution for 0.5 mole fraction, but the distribution had a markedly larger constant term for the two biased transfections.

Thus, the biased expression levels generate a component of CV that does not decrease with expression levels. This extra variation has an unknown source. We note that biased transfections are expected to produce an excess of unpaired donor or acceptor fluorophores, depending on the subunit ratio. Therefore part of the excess variation may arise from pixel to pixel variations in *f_D_*^′^ or in *e*, a parameter related to variations in $\sqrt{I_{A}}$ (Supplementary Figure S2).

### 2.3. Pharmacological Chaperoning Stabilizes Alternative α4β2 Stoichiometries

We have also studied pharmacological manipulations thought to produce changes in the subunit stoichiometry [6,18,27]. Our experiments have typically utilized the cDNA ratio of 0.5 (as in the experiments of Figure 3, middle panels). Pharmacological chaperoning is expected to produce less complete changes in stoichiometry than the biased cDNA ratios. As expected, the pharmacological chaperones have generally produced $\bar{NFRET}$ averages different from the cells treated with no drug (Figure 4), but intermediate between the extremes of Figure 3. Nicotine produced a lower $\bar{NFRET}$, consistent with many previous studies that nicotine stabilizes the (α4)_2_(β2)_3_ stoichiometry. On the other hand, cytisine increased $\bar{NFRET}$ [27]. This finding, novel at the time, has since been confirmed by single-molecule measurements of nAChRs on the plasma membrane, using zero-mode waveguides [27]. Clearly, cytisine stabilizes the (α4)_3_(β2)_2_ stoichiometry.

From these pharmacological experiments, we have analyzed the pixel-by-pixel data [27]. This analysis, similar to that of Figure 3, extracted the values of $\bar{NFRET_{low}},\bar{NFRET_{high}}$ for the two Gaussian components, which correspond to (α4)_2_(β2)_3_ and (α4)_3_(β2)_2_ nAChRs, respectively. The ratio $\bar{NFRET_{high}}/\bar{NFRET_{low}}$ is usually similar to the values obtained with biased transfections [27]. The values of *W_high_* = *Int_high_*/(*Int_low_* + *Int_high_*) for the untreated cells were similar to those of Figure 3: for nicotine, less than the untreated cells; and for cytisine, more than the untreated cells. That nicotine and cytisine respectively decrease and increase *W_high_*, compared with untreated cells, was observed in each of five independent transfections like that of Figure 4. The values of *W_high_* produced by the pharmacological chaperones are less extreme than those for biased transfections, again indicating that pharmacological chaperoning by nicotine and cytisine partially shifts the population toward either stoichiometry.

### 2.4. One *vs*. Two β2 < α4 Subunit Interfaces

The data do not allow for a choice between subunit orders that involve one *vs*. two β2 < α4 subunit interfaces; these interfaces are also thought to be high-affinity ligand binding sites. The two-interface model is supported by two facts about α4β2 nAChRs: the Hill coefficient for the dose-response relation exceeds unity; and concatameric subunits function well when there are two β2 < α4 units within a pentamer [35]. Nonetheless, other Cys-loop receptors (5-HT_3_, GABA_A_, glycine, and GluCl channels) may display different subunit orders. All theory and analysis in the paper assumes that only two populations are present (those pictured in Figure 1A). Supplementary Materials provides simulations for the single-interface model, but again for only two populations.

### 2.5. Requirements for the Procedure

We outline the optimal pre-requisites for the procedures. First, one requires some assurance that the fluorophores do not markedly distort function. For the present fluorescent constructs, several studies provide this assurance [6,16–18,29,36]. It is now also possible to achieve functional responses when the fluorescent proteins are placed at the extracellular *C*-terminus [37].

One requires a modest density of receptors. We estimate that the nAChRs in our studies are present at a density of 3–100 μm^−2^, because we have utilized similar cells and microscopes for single-molecule fluorescence [38–40]. Much lower densities would provide insufficient signals. Much higher densities could produce “stochastic” FRET.

One would prefer a system to determine the NFRET values of nearly pure stoichiometries. This allows one to define the NFRET values corresponding to *Ē*_3,2_*, Ē*_2,3_ directly, as in Figure 2C. We have accomplished this via transient transfections with biased cDNA ratios. In the absence of this preferred route, one can extract the appropriate NFRET values from Gaussian fits to NFRET distributions. As shown in Figures 3 and 4, the Gaussian fits produce a $\bar{NFRET_{high}}/\bar{NFRET_{low}}$ value corresponding well to directly determined $\bar{NFRET}$.

## 3. Conclusions

These experiments analyze FRET-based measurements of changed nAChR stoichiometry in live cells with good spatial resolution. We emphasize the detection of altered stoichiometry rather than absolute values. Nonetheless, it is rather satisfactory that the experimental values of $\bar{NFRET_{high}}/\bar{NFRET_{low}}$ are simulated by plausible parameters for the structure of α4β2 nAChRs. It is also satisfactory that consistent conclusions arise from two different analyses of the data: $\bar{NFRET}$, and *W_high_*. The values for net FRET, *Ē*_3,2_ / *Ē*_2,3_*,* follow the same trends (Supplementary Information). Summarizing these conclusions: the biased subunit transfections favor the two stoichiometries in the expected directions; pharmacological chaperoning by nicotine favors the (α4)_2_(β2)_3_ stoichiometry; and cytisine favors the (α4)_3_(β2)_2_ stoichiometry [2,41].

The present simulations and experiments incorporate donors or acceptors within all receptor subunits. This tactic provides the largest possible fluorescence signals [42], roughly twice as large as in our previous quantitative FRET methods, where donor and acceptor fluorophores were included only in α4 subunits, or only in β2 subunits [6,18].

### 3.1. Investigations that Might Use the Procedures

The experiments produce valid results independently of knowledge about *R_1,ave_*. This is fortunate, because the intracellular domains of Cys-loop receptors have not been characterized structurally, despite the excellent progress on structures for the extracellular binding regions and transmembrane domains [43–45]. We emphasize that distance measurements are not the focus of this study; rather, we describe useful experimentally determined FRET-related measurements that reveal changes in stoichiometry.

Which range of *R_1,ave_* values would yield useful data? Because the fluorescent proteins are cylinders with diameter of 25 Å and length 40 Å [46], they could physically approach as close as *R_1,ave_*~25 Å, which is the lower limit of our simulations (Figure 2A) but predicts *Ē*_3,2_ / *Ē*_2,3_ and *NFRET*_3,2_/*NFRET*_2,3_ values markedly lower than we measure. At the other extreme, *R_1,ave_* might be ~40 Å greater than the intersubunit distance of the residues at the point of insertion; but *R_1,ave_* > ~70 Å would probably give FRET values below the measurable range.

The Introduction summarizes the various pharmacological and electrophysiological characteristics that depend on the subunit stoichiometry. It is also possible to employ the pixel-based resolution in order to distinguish between nAChR stoichiometry in distinct organelles, providing a cell biological basis for the partially distinct pixel distributions. For instance, we concluded that the GABA transporter GAT1 exists in different multimerization states in perinuclear *vs*. near-plasma membrane regions [19]. The resolution is poorer than the 69 nm square represented by a pixel; therefore one should remember that adjacent pixels are highly correlated. Because the stoichiometry is determined in the ER, but governs exit from the ER as well as trafficking through the secretory pathway to the membrane, the procedures developed here will aid in several investigations. We showed that the FRET procedures can distinguish stoichiometry changes during chronic exposure to nicotinic ligands [5,6]. Stoichiometry may also change with expression of auxiliary subunits or chaperone proteins [12], with mutations causing autosomal dominant nocturnal frontal lobe epilepsy [18], and with mutations of ER retention and export motifs [6]. Some of these manipulations also affect the efficiency of nAChR exit from the ER [6]. The procedures described here should be applied in additional studies.

### 3.2. Other Cys-Loop Receptors

We expect most Cys-loop receptors to be characterized by a geometry factor nearer to 1.62 and by an asymmetry factor nearer to zero, compared with the α4β2 receptors, for the reason that intracellular M3–M4 loop lengths of other Cys-loop receptors occupy a more uniform distribution. Thus the analysis presented here may be generally applied.

## Supplementary Materials



## Abbreviations

EGFP: enhanced green fluorescent protein
ER: endoplasmic reticulum
FRET: Förster resonance energy transfer
nAChR: neuronal nicotinic acetylcholine receptors
mCherry: monomeric cherry fluorescent protein
NFRET: normalized Förster resonance energy transfer

## References

[1] Zwart R., Vijverberg H.P. (1998). Four pharmacologically distinct subtypes of α4β2 nicotinic acetylcholine receptor expressed in *Xenopus laevis* oocytes. Mol. Pharmacol.

[2] Moroni M., Zwart R., Sher E., Cassels B.K., Bermudez I. (2006). α4β2 nicotinic receptors with high and low acetylcholine sensitivity: Pharmacology, stoichiometry, and sensitivity to long-term exposure to nicotine. Mol. Pharmacol.

[3] Xiu X., Puskar N.L., Shanata J.A., Lester H.A., Dougherty D.A. (2009). Nicotine binding to brain receptors requires a strong cation-π interaction. Nature.

[4] Tapia L., Kuryatov A., Lindstrom J. (2007). Ca^2+^ permeability of the (α4)_3_(β2)_2_ stoichiometry greatly exceeds that of (α4)_2_(β2)_3_ human acetylcholine receptors. Mol. Pharmacol.

[5] Nelson M.E., Kuryatov A., Choi C.H., Zhou Y., Lindstrom J. (2003). Alternate stoichiometries of α4β2 nicotinic acetylcholine receptors. Mol. Pharmacol.

[6] Srinivasan R., Pantoja R., Moss F.J., Mackey E.D.W., Son C., Miwa J., Lester H.A. (2011). Nicotine upregulates α4β2 nicotinic receptors and ER exit sites via stoichiometry-dependent chaperoning. J. Gen. Physiol.

[7] Commons K.G. (2008). α4 containing nicotinic receptors are positioned to mediate postsynaptic effects on 5-HT neurons in the rat dorsal raphe nucleus. Neuroscience.

[8] Shapiro M.S., Hille B. (1993). Substance P and somatostatin inhibit calcium channels in rat sympathetic neurons via different G protein pathways. Neuron.

[9] Sallette J., Pons S., Devillers-Thiery A., Soudant M., de Carvalho L.P., Changeux J.P., Corringer P.J. (2005). Nicotine upregulates its own receptors through enhanced intracellular maturation. Neuron.

[10] Kuryatov A., Luo J., Cooper J., Lindstrom J. (2005). Nicotine acts as a pharmacological chaperone to up-regulate human α4β2 acetylcholine receptors. Mol. Pharmacol.

[11] Lester H.A., Xiao C., Srinivasan R., Son C., Miwa J., Pantoja R., Dougherty D.A., Banghart M.R., Goate A.M., Wang J.C. (2009). Nicotine is a selective pharmacological chaperone of acetylcholine receptor number and stoichiometry. Implications for drug discovery. AAPS J.

[12] Miwa J.M., Freedman R., Lester H.A. (2011). Neural systems governed by nicotinic acetylcholine receptors: Emerging hypotheses. Neuron.

[13] Gopalakrishnan M., Molinari E.J., Sullivan J.P. (1997). Regulation of human α4β2 neuronal nicotinic acetylcholine receptors by cholinergic channel ligands and second messenger pathways. Mol. Pharmacol.

[14] Whiteaker P., Sharples C.G., Wonnacott S. (1998). Agonist-induced up-regulation of α4β2 nicotinic acetylcholine receptors in M10 cells: Pharmacological and spatial definition. Mol. Pharmacol.

[15] Kishi M., Steinbach J.H. (2006). Role of the agonist binding site in up-regulation of neuronal nicotinic α4β2 receptors. Mol. Pharmacol.

[16] Nashmi R., Dickinson M.E., McKinney S., Jareb M., Labarca C., Fraser S.E., Lester H.A. (2003). Assembly of α4β2 nicotinic acetylcholine receptors assessed with functional fluorescently labeled subunits: Effects of localization, trafficking, and nicotine-induced upregulation in clonal mammalian cells and in cultured midbrain neurons. J. Neurosci.

[17] Drenan R.M., Nashmi R., Imoukhuede P.I., Just H., McKinney S., Lester H.A. (2008). Subcellular trafficking, pentameric assembly and subunit stoichiometry of neuronal nicotinic ACh receptors containing Fluorescently-Labeled α6 and β3 subunits. Mol. Pharmacol.

[18] Son C.D., Moss F.J., Cohen B.N., Lester H.A. (2009). Nicotine normalizes intracellular subunit stoichiometry of nicotinic receptors carrying mutations linked to autosomal dominant nocturnal frontal lobe epilepsy. Mol. Pharmacol.

[19] Moss F.J., Imoukhuede P.I., Scott K., Hu J., Jankowsky J.L., Quick M.W., Lester H.A. (2009). GABA transporter function, oligomerization state, and anchoring: Correlates with subcellularly resolved FRET. J. Gen. Physiol.

[20] Corry B., Jayatilaka D., Rigby P. (2005). A flexible approach to the calculation of resonance energy transfer efficiency between multiple donors and acceptors in complex geometries. Biophys. J.

[21] Corry B., Jayatilaka D., Martinac B., Rigby P. (2006). Determination of the orientational distribution and orientation factor for transfer between membrane-bound fluorophores using a confocal microscope. Biophys. J.

[22] Raicu V. (2007). Efficiency of resonance energy transfer in homo-oligomeric complexes of proteins. J. Biol. Phys.

[23] Deplazes E., Jayatilaka D., Corry B. (2012). ExiFRET: Flexible tool for understanding FRET in complex geometries. J. Biomed. Opt.

[24] Wlodarczyk J., Woehler A., Kobe F., Ponimaskin E., Zeug A., Neher E. (2008). Analysis of FRET signals in the presence of free donors and acceptors. Biophys. J.

[25] Akrap N., Seidel T., Barisas B.G. (2010). Forster distances for fluorescence resonant energy transfer between mCherry and other visible fluorescent proteins. Anal. Biochem.

[26] Wolber P.K., Hudson B.S. (1979). An analytic solution to the Forster energy transfer problem in two dimensions. Biophys. J.

[27] Srinivasan R., Richards C.I., Xiao C., Rhee D., Pantoja R., Dougherty D.A., Miwa J.M., Lester H.A. (2012). Pharmacological chaperoning of nicotinic acetylcholine receptors reduces the ER stress response. Mol. Pharmacol.

[28] Hoppe A., Christensen K., Swanson J.A. (2002). Fluorescence resonance energy transfer-based stoichiometry in living cells. Biophys. J.

[29] Nashmi R., Xiao C., Deshpande P., McKinney S., Grady S.R., Whiteaker P., Huang Q., McClure-Begley T., Lindstrom J.M., Labarca C. (2007). Chronic nicotine cell specifically upregulates functional α4* nicotinic receptors: Basis for both tolerance in midbrain and enhanced long-term potentiation in perforant path. J. Neurosci.

[30] Raicu V., Stoneman M., Fung R., Melnichuk M., Jansma D., Pisterzi L., Rath S., Fox M., Wells J., Saldin D. (2009). Determination of supramolecular structure and spatial distribution of protein complexes in living cells. Nat. Photonics.

[31] Singh D.R., Raicu V. (2010). Comparison between whole distribution- and average-based approaches to the determination of fluorescence resonance energy transfer efficiency in ensembles of proteins in living cells. Biophys. J.

[32] Tadross M.R., Park S.A., Veeramani B., Yue D.T. (2009). Robust approaches to quantitative ratiometric FRET imaging of CFP/YFP fluorophores under confocal microscopy. J. Microsc.

[33] Xia Z., Liu Y. (2001). Reliable and global measurement of fluorescence resonance energy transfer using fluorescence microscopes. Biophys. J.

[34] Kuryatov A., Onksen J., Lindstrom J. (2008). Roles of accessory subunits in α4β2α5 nicotinic receptors. Mol. Pharmacol.

[35] Zhou Y., Nelson M.E., Kuryatov A., Choi C., Cooper J., Lindstrom J. (2003). Human α4β2 acetylcholine receptors formed from linked subunits. J. Neurosci.

[36] Khakh B.S., Fisher J.A., Nashmi R., Bowser D.N., Lester H.A. (2005). An angstrom scale interaction between plasma membrane ATP-gated P2×2 and α4β2 nicotinic channels measured with FRET and TIRF microscopy. J. Neurosci.

[37] Richards C.I., Srinivasan R., Xiao C., Mackey E.D.W., Miwa J.M., Lester H.A. (2011). Trafficking of α4* nicotinic receptors revealed by superecliptic phluorin: Effects of a β4 ALS-associated mutation and chronic exposure to nicotine. J. Biol. Chem.

[38] Chiu C.S., Kartalov E., Unger M., Quake S., Lester H.A. (2001). Single-molecule measurements calibrate green fluorescent protein surface densities on transparent beads for use with ‘knock-in’ animals and other expression systems. J. Neurosci. Methods.

[39] Chiu C.S., Jensen K., Sokolova I., Wang D., Li M., Deshpande P., Davidson N., Mody I., Quick M.W., Quake S.R. (2002). Number, density, and surface/cytoplasmic distribution of GABA transporters at presynaptic structures of knock-in mice carrying GABA transporter subtype 1-green fluorescent protein fusions. J. Neurosci.

[40] Pantoja R., Rodriguez E.A., Dibas M.I., Dougherty D.A., Lester H.A. (2009). Single-molecule imaging of a fluorescent unnatural amino Acid incorporated into nicotinic receptors. Biophys. J.

[41] Richards C.I., Luong K., Srinivasan R., Turner S.W., Dougherty D.A., Korlach J., Lester H.A. (2012). Live-cell imaging of single receptor composition using zero-mode waveguides nanostructures. Nano Lett.

[42] Fabian A.I., Rente T., Szollosi J., Matyus L., Jenei A. (2010). Strength in numbers: Effects of acceptor abundance on FRET efficiency. Chemphyschem.

[43] Brejc K., van Dijk W.J., Klaassen R.V., Schuurmans M., van der Oost J., Smit A.B., Sixma T.K. (2001). Crystal structure of an ACh-binding protein reveals the ligand-binding domain of nicotinic receptors. Nature.

[44] Unwin N. (2005). Refined structure of the nicotinic acetylcholine receptor at 4A resolution. J. Mol. Biol.

[45] Hibbs R.E., Gouaux E. (2011). Principles of activation and permeation in an anion-selective Cys-loop receptor. Nature.

[46] Ormo M., Cubitt A.B., Kallio K., Gross L.A., Tsien R.Y., Remington S.J. (1996). Crystal structure of the *Aequorea victoria* green fluorescent protein. Science.

