# Supplementary material for: Förster Resonance Energy Transfer (FRET) Correlates of Altered Subunit Stoichiometry in Cys-Loop Receptors, Exemplified by Nicotinic α4β2
Source: Int J Mol Sci. 2012 Aug 10;13(8):10022–40. doi: 10.3390/ijms130810022 (PMC3431844; doi:10.3390/ijms130810022)
Supplement: Supplementary file 1 [file ijms-13-10022-s001.pdf]

Förster Resonance Energy Transfer (FRET) Correlates of Altered Subunit Stoichiometry in Cys-Loop Receptors, Exemplified by Nicotinic α4β2

Supplementary Information

1. Single β2 < α4 Interface

Figure S1. presents calculations for a subunit order containing only a single β2<α4 interface. The top panel presents a table of $n_{i,j}$ values extending Figure 1D, and including the $n_{i,j}$ values for the single-interface structure. The equations for $\overline{E}_{3,2}/\overline{E}_{2,3}$ and $NFRET_{3,2}$ / $NFRET_{2,3}$ are derived by analogy to Equations 2 through 9. Panels A through D present the results. For this model, the simulations depend more strongly on the geometry factor G than on the asymmetry factor Δ; therefore plots with G on the *y*-axis are presented in C and D.

	Two β2<α4 interfaces		Single β2<α4 interface	
	1 23<br>β<αββ<α	1 2<br>β<αβ<αα	123<br>βββ<αα	12<br>ββ<ααα
	"n-factors" that equal 1 (all others = 0)			
**β2 #1**	$n_{1,-1}$, $n_{1,+1}$	$n_{1,-2}$, $n_{1,-1}$, $n_{1,+1}$	$n_{1,-2}$, $n_{1,-1}$	$n_{1,-2}$, $n_{1,-1}$, $n_{1,+2}$
**β2 #2**	$n_{2,-1}$, $n_{2,+2}$	$n_{2,-1}$, $n_{2,+1}$, $n_{2,+2}$	$n_{2,-2}$, $n_{2,+2}$	$n_{2,-2}$, $n_{2,+1}$, $n_{2,+2}$
**β2 #3**	$n_{,3,-2}$, $n_{3,+1}$		$n_{3,+1}$, $n_{3,+2}$	

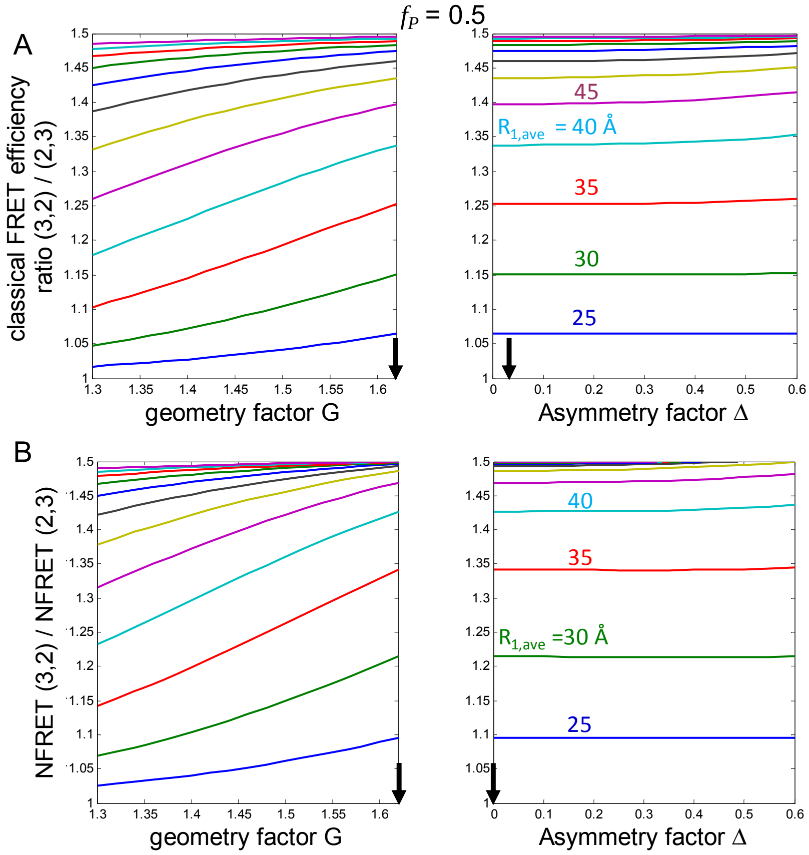

Figure S1. *Cont.*

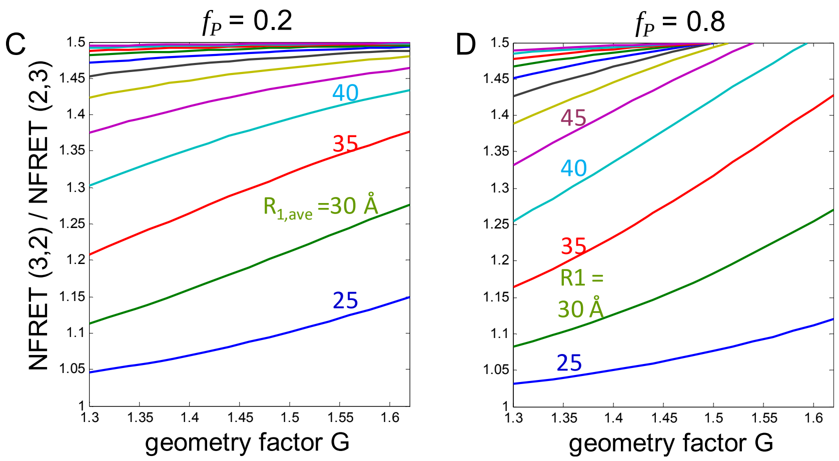

2. Stoichiometry-Dependent Changes in I_A or $f_D^{'}$

Distortions in $NFRET_{3,2} / NFRET_{2,3}$ could arise from factors that render I_A different for the two stoichiometries. We have not systematically studied this possibility; but it should be considered in view of the reports about dead-end intermediates in assembly of nAChRs [1,2], as well as reports that degradation could occur differentially for the two stoichiometric forms [3].

To simulate an effect of stoichiometry on I_A, we define the parameter *e*, which increases I_A for the three-acceptor stoichiometry more than for the two-acceptor stoichiometry. We write, for the $(\alpha4)_2(\beta2)_3$ stoichiometry,

$$I_A = (1-e) + 3e;$$

for the $(\alpha4)_3(\beta2)_2$ stoichiometry,

$$I_A = (1-e) + 2e.$$

Figure S2 presents the calculations for $NFRET_{3,2} / NFRET_{2,3}$ resulting from the assumption that *e* = 0.2. Evidently the major result is to decrease the expected measured $NFRET_{3,2} / NFRET_{2,3}$ ratios. If the sign of *e* is reversed, the expected $NFRET_{3,2} / NFRET_{2,3}$ ratios increase. Thus, e = −0.2 predicts $NFRET_{3,2} / NFRET_{2,3}$ > 1.2 for all $R_{1,ave}$> ~25 Å (not shown), or essentially outside the measured range of data.

Variations in $f_D^{'}$ could arise as follows. If $f_P = 0.5$, then $1-(1-f_P)^2 = 75\%$ of donors in $(\alpha4)_2(\beta2)_3$ nAChRs participate in FRET; but $1-(1-f_P)^3 = 87.5\%$ of donors in $(\alpha4)_3(\beta2)_2$ nAChRs participate in FRET. This would affect the $\sqrt{I_D}$ factor in Equation 8 and would be simulated with a term containing *e*, similar to those above. The expected effects would be of similar magnitude to those of Figure S2.

Figure S2. Calculations for $NFRET_{3,2}$ / $NFRET_{2,3}$ resulting from the assumption that $e = 0.2$.

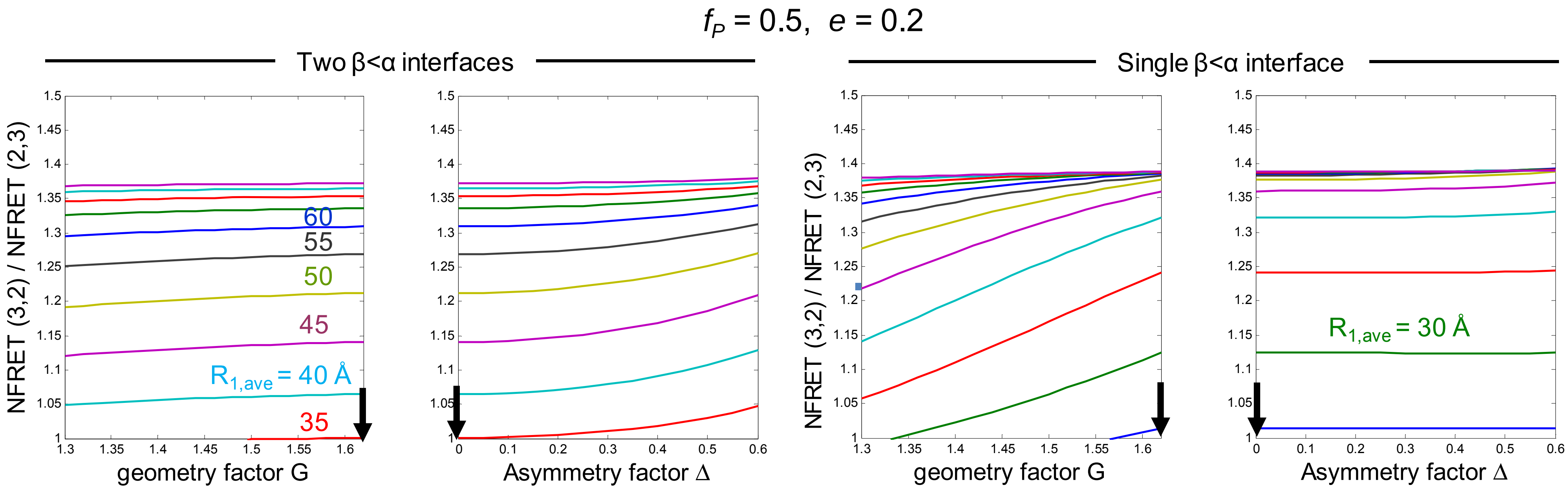

3. Net FRET Analyses

Measurements of net FRET utilize I_{DA}, uncorrected for total donor or acceptor intensity. Figure S3 presents net FRET distributions for the two manipulations of this study: biased transfections and 48 h drug treatment. The untransformed data are evidently not suited for analysis by statistics that assume normal distributions. We have therefore simply computed the mean across all pixels. The mean net FRET values do follow the same trend of the values for $\overline{NFRET}$, and for W_{high}. The lowest values are recorded for the higher mole fraction of β2 cDNA and for incubation in nicotine; the highest values are recorded for the lower mole fraction of β2 cDNA and for incubation in cytisine. In the biased subunit experiment, the ratio between lowest and highest values for net FRET (1.29) is quite consistent with the values simulated for inter-fluorophore distances of 35–50 Å. In the pharmacological chaperoning experiment, the analogous value is 1.27, not markedly different. Evidently the net FRET measurements lack the power to resolve the subtle differences resolved in the NFRET experiments. Also note that, for the identical conditions (0.5 mole fraction β2 cDNA, no drug treatment), the absolute values of the net FRET means differ between the two experiments, as expected for variations among cell batches, transfection efficiency, and other factors; these variables are largely eliminated in NFRET experiments. Thus the net FRET analyses, while modestly reinforcing the qualitative conclusions about stoichiometry observed in the NFRET experiments, are less useful than the latter.

Figure S3. Pixel distributions for net FRET in experiments that vary (**A**) mole fraction β2 mRNA or (**B**) 48 h drug treatment. Each sample had >10^6 pixels.

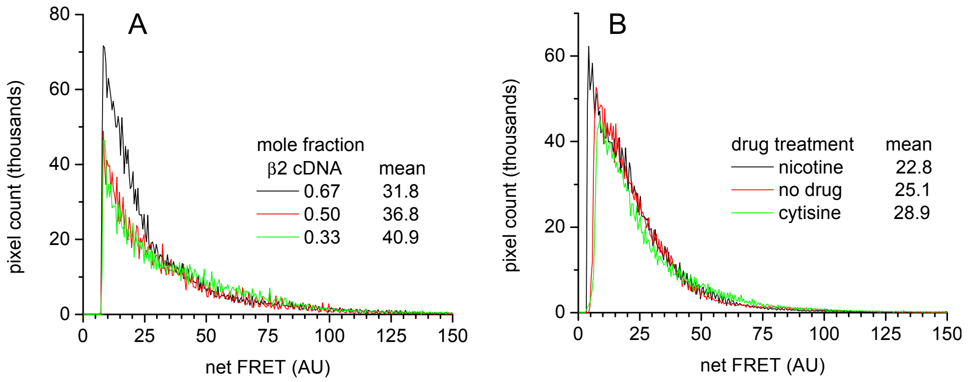

References

1. Nelson, M.E.; Kuryatov, A.; Choi, C.H.; Zhou, Y.; Lindstrom, J. Alternate stoichiometries of α4β2 nicotinic acetylcholine receptors. *Mol. Pharmacol.* **2003**, *63*, 332–341.
2. Kuryatov, A.; Luo, J.; Cooper, J.; Lindstrom, J. Nicotine acts as a pharmacological chaperone to up-regulate human α4β2 acetylcholine receptors. *Mol. Pharmacol.* **2005**, *68*, 1839–1851.
3. Srinivasan, R.; Pantoja, R.; Moss, F.J.; Mackey, E.D.W.; Son, C.; Miwa, J.; Lester, H.A. Nicotine upregulates α4β2 nicotinic receptors and ER exit sites via stoichiometry-dependent chaperoning. *J. Gen .Physiol.* **2011**, *137*, 59–79.
